# Sulfur Dioxide Activates Cl^-^/HCO_3_^-^ Exchanger via Sulphenylating AE2 to Reduce Intracellular pH in Vascular Smooth Muscle Cells

**DOI:** 10.3389/fphar.2019.00313

**Published:** 2019-03-27

**Authors:** Yi Wang, Xiuli Wang, Selena Chen, Xiaoyu Tian, Lulu Zhang, Yaqian Huang, Chaoshu Tang, Junbao Du, Hongfang Jin

**Affiliations:** ^1^Department of Pediatrics, Peking University First Hospital, Beijing, China; ^2^Division of Biological Sciences, University of California, San Diego, La Jolla, CA, United States; ^3^Department of Physiology and Pathophysiology, Peking University Health Science Center, Beijing, China; ^4^Key Laboratory of Molecular Cardiology, Ministry of Education, Beijing, China

**Keywords:** sulfur dioxide, vascular smooth muscle cell, intracellular pH, Cl^-^/HCO_3_^-^ exchanger, AE2 sulphenylation

## Abstract

Sulfur dioxide (SO_2_) is a colorless and irritating gas. Recent studies indicate that SO_2_ acts as the gas signal molecule and inhibits vascular smooth muscle cell (VSMC) proliferation. Cell proliferation depends on intracellular pH (pH_i_). Transmembrane cystein mutation of Na^+^- independent Cl^-^/HCO_3_^-^ exchanger (anion exchanger, AE) affects pH_i_. However, whether SO_2_ inhibits VSMC proliferation by reducing pH_i_ is still unknown. Here, we investigated whether SO_2_ reduced pH_i_ to inhibit the proliferation of VSMCs and explore its molecular mechanisms. Within a range of 50–200 μM, SO_2_ was found to lower the pH_i_ in VSMCs. Concurrently, NH_4_Cl pre-perfusion showed that SO_2_ significantly activated AE, whereas the AE inhibitor 4,4′-diisothiocyanatostilbene- 2,20-disulfonic acid (DIDS) significantly attenuated the effect of SO_2_ on pH_i_ in VSMCs. While 200 μM SO_2_ sulphenylated AE2, while dithiothreitol (DTT) blocked the sulphenylation of AE2 and subsequent AE activation by SO_2_, thereby restoring the pH_i_ in VSMCs. Furthermore, DIDS pretreatment eliminated SO_2_-induced inhibition of PDGF-BB-stimulated VSMC proliferation. We report for the first time that SO_2_ inhibits VSMC proliferation in part by direct activation of the AE via posttranslational sulphenylation and induction of intracellular acidification.

## Introduction

Aberrant proliferation of vascular smooth muscle cells (VSMCs) contributes to the pathological change of vascular diseases such as hypertension, diabetic angiopathy and atherosclerosis (Owens et al., 2004; Chistiakov et al., 2015; Bennett et al., 2016). Previous studies have shown that the intracellular pH (pH_i_) is an important factor involved in the regulation of cell proliferation. Additionally, cell proliferation of multiple species is dependent on the pH_i_. Mitogen stimulation promotes cell cycle progression and ultimately proliferation. DNA, RNA and protein synthesis all require an alkaline pH_i_ (Schreiber, 2005; Flinck et al., 2018).

To maintain pH_i_ homeostasis, cells utilize ionophores on the membrane to regulate the pH_i_ within a narrow physiological range (Cardone et al., 2005; Boron et al., 2009; Casey et al., 2010). Those ionophores include channels, pumps, exchangers and cotransporters, all of which synergistically regulate the influx and outflux of H^+^/HCO_3_^-^ ions (Concepcion et al., 2013; Chen et al., 2018). Among them, the Na^+^-independent and electroneutral Cl^-^/HCO_3_^-^ exchanger (anion exchanger, AE) is encoded by the *SLC4* gene family, including *SLC4A1/AE1, SLC4A2/AE2*, and *SLC4A3/AE3*, which help regulate the pH_i_, cell volume and membrane potential of various cell types (Alper, 2009; Liu et al., 2015). Genome-wide association analysis showed that *AE2* exon deletion resulted in a loss of function of AE2 in osteoclast and cell alkalization, resulting in bone resorption lacunae disorder, the genetic cause of Angoras cattle and mouse osteopetrosis (Meyers et al., 2010; Coury et al., 2013). Transforming growth factor beta 1 promotes fibroblast cell membrane AE2 expression and HCO_3_^-^ excretion, which can neutralize tumor microenvironment H^+^ ions to inhibit tumor cell invasion (Hulikova et al., 2016). Concepcion et al. (2014) found that compared with wild-type mice, the pH_i_ of CD8^+^ T cells derived from *AE2* knockout mice is significantly increased, and CD8^+^ T cell proliferation and activation levels are obviously enhanced after CD3 stimulation.

Reimold et al. (2013) constructed a mouse AE2 model devoid of transmembrane domain cysteine (Cys) residues to investigate structure-function relationships for AE2. They found that extracellular pH was alkaline-shifted by a minimum of 0.6-0.7 pH units, and the anion exchange rate was significantly decreased in the absence of transmembrane domain Cys residues (Reimold et al., 2013). Sulfur dioxide (SO_2_) was found to oxidize the -SH of Cys to -SOH, which changed the protein conformation and affected protein activity levels (Svoboda et al., 2012). We confirmed that SO_2_ suppressed the inflammatory response by sulphenylating NF-κB p65 at Cys^38^ in oleic acid-induced acute lung injury (Chen et al., 2017).

Recent studies have shown that aspartate aminotransferase can be catalyzed and produces SO_2_ in the metabolic pathway of sulfur-containing amino acids in mammalian organisms. Furthermore, SO_2_, which is considered the fourth gas signal molecule followed by NO, CO and H_2_S, plays an important role in the regulation of cardiovascular physiology and pathophysiology, such as vasodilation, inhibition of vascular calcification and inflammation, anti-oxidation and protection of the myocardium (Du et al., 2008; Sun et al., 2010; Zhang et al., 2011; Huang et al., 2016; Li et al., 2016; Yu et al., 2018).

Therefore, we hypothesized that SO_2_ might activate the AE in VSMCs, thereby lowering the pH_i_ and further inhibiting VSMC proliferation. It has been reported that AE expression varies significantly among different tissues, and only *SLC4A2/AE2* mRNA expression is detected in the VSMC cell line A7r5 cells (Brosius et al., 1997). Therefore, in this study, we aimed to determine whether SO_2_ impacts the pH_i_ and further inhibits the proliferation of VSMCs, as well as explore underlying molecular mechanisms.

## Materials and Methods

### Chemicals and Drugs

Sodium sulfite and sodium bisulfite (Na_2_SO_3_/NaHSO_3_), 4,4′- diisothiocyanatostilbene -2,20-disulfonic acid (DIDS) and H89 were purchased from Sigma-Aldrich (St. Louis, MO, United States). Nigericin (N1495) and Fluo 4-AM (F14217) were purchased from Invitrogen (Eugene, OR, United States), and BCECF/AM was purchased from Thermo Scientific (Waltham, MA, United States). Dithiothreitol (DTT) was purchased from Roche Diagnostics GmbH (Mannheim, Germany). Bay K8644 was purchased from selleck (Houston, TX, United States). SO_2_ donor was freshly prepared using Na_2_SO3/NaHSO_3_ dissolved in deionized water in 3:1 mole ratio. DIDS, H89, BCECF/AM and Fluo 4-AM were dissolved in DMSO. Nigericin was dissolved in ethanol.

### Cells and Cell Culture

A7r5 VSMCs were purchased from Kunming Cell Bank of Chinese Academy of Sciences (Kunming, China). Cell culture refers to previous literature (Liu et al., 2014). The cells were cultured in Dulbecco’s modified Eagle’s medium (DMEM) supplemented with 10% fetal bovine serum and 1% antibiotics (penicillin and streptomycin), and cells were placed in an incubator containing 5% CO_2_ at 37°C. To detect the pH_i_, cells were seeded in confocal dishes and experiments were performed once the cell density reached 90%.

### Measurement of pH_i_ in VSMCs

Measurement of pH_i_ was performed according to the literature (Lee et al., 2007; Galifianakis et al., 2018). Fluorescent indicator BCECF/AM was used to monitor pH_i_ changes. Cells were washed twice with Krebs’ bicarbonate buffer, followed by the incubation with Krebs’ buffer containing 0.5 μM BCECF/AM for 30 min at room temperature. Loaded cells were washed twice with fresh Krebs’ buffer to remove unbound dye and left at room temperature for another 30 min to allow the dye to be fully de-esterified in the cells. The pH_i_ was monitored using a confocal scanning laser microscope (Leica TCS SP8 MP FLIM, Mannheim, Baden-Württember, Germany). The fluorescence intensity was measured at an excitation wavelength of 405 and 496 nm, and an emission wavelength of 535 nm was recorded. The fluorescence intensity ratio (F496/F405) was used to evaluate the pH_i_. pH_i_ image analysis was performed using LAF-AS software (Leica). The Krebs’ buffer was prepared as follows (mM): 118 NaCl, 5.4 KCl, 1.3 CaCl_2_, 1.2 MgSO_4_, 1.2 KH_2_PO_4_, 25 NaHCO_3_ and 11.7 glucose, and the pH was adjusted to 7.4 with NaOH. During the measurement of pH_i_, drugs were added directly to the buffer and the fluorescence intensity was recorded. A high KCl solution (130 mM KCl, 10 mM Hepes buffer, pH 6.3–9.1) was prepared, and H^+^ was equilibrated with 20 μM cation ionophore nigericin to prepare a fluorescence intensity-pH_i_ standard curve (Supplementary Figure S1). The different concentrations of SO_2_ in the study of pH_i_ were grouped into control, SO_2_ (50 μM), SO_2_ (100 μM), and SO_2_ (200 μM). The change of pH_i_ was calculated as the difference in pH_i_ between the starting time point of SO_2_ treatment and 9 min after SO_2_ treatment.

### Determination of AE Activity in VSMCs

AE activity was measured according to the method described in the previous literature (Simchowitz and roos, 1985; Xu and Spitzer, 1994; Lee et al., 2007). In brief, the activity of AE in A7r5 cells was evaluated by detecting the recovery rate of cells from an intracellular alkalinization. Cells were loaded with BCECF/AM as previously described. The Hepes buffer solution was prepared as follows (mM): 150 NaCl, 5.4 KCl, 2 CaCl_2_, 1 MgCl_2_, 5 Hepes and 10 glucose, adjusted to pH 7.4 with NaOH. At the first min, 20 mM NH_4_Cl was quickly added to the buffer. Once the cells were exposed to NH_4_Cl, NH_3_ rapidly diffused into the cells and combined with intracellular H^+^ to form NH_4_^+^, leading to rapid intracellular alkalinization. The pH_i_ gradually decreases from the alkaline peak as HCO_3_^-^ ions efflux during AE stimulation. The rate at which the pH_i_ returns within the first minute (ΔpH/min) represents AE activity. For the SO_2_ and the AE activation experiment, the groups were divided as follows: control, SO_2_ (100 μM) and SO_2_ (200 μM), and SO_2_ donor administrated 10 min before pH_i_ measurement. To verify that SO_2_ stimulated AE via sulphenylation of AE2, we divided the groups as follows: control, SO_2_, SO_2_ + DTT or PDGF-BB, PDGF-BB + SO_2_ and PDGF-BB + SO_2_ + DTT. One hour before the pH_i_ measurement, cells were treated with 50 ng/ml PDGF-BB, and 50 min before pH_i_ measurement, cells were pretreated with 200 μM SO_2_ and / or 0.4 mM DTT.

### Western Blotting

Vascular smooth muscle cells were seeded in six-well plates and upon a cell density of 60–70%, were synchronized with DMEM containing 0.5% FBS for 24 h.

To confirm that AE was involved in the process of SO_2_ inhibition of PDGF-BB-induced VSMC proliferation, cell were either (1) untreated; (2) treated with 50 ng/ml PDGF-BBC for 24 h; (3) pretreated with 200 μM SO_2_ donor for 30 min, then 50 ng/ml PDGF-BB for 24 h; (4) treated with 200 μM SO_2_ donor for 30 min; (5) treated with 50 ng/ml PDGF-BB and 30 μM DIDS for 24 h; and (6) pretreated with 200 μM SO_2_ donor for 30 min, then 50 ng/ml PDGF-BB and 30 μM DIDS for 24 h.

To measure the phosphorylation level of PKA by SO_2_, cells were either (1) untreated; (2) treated with 20 μM H89 for 30 min; (3) pretreated with 20 μM H89 for 30 min, and 200 μM SO_2_ donor for 10 min; or (4) treated with 200 μM SO_2_ donor for 10 min.

All cells were collected and lysed in lysis buffer (50 mM Tris-HCl [pH 7.4], 150 mM NaCl, 1 mM EDTA, 1% NP40, 0.25% sodium deoxycholate, 1 mM PMSF, protease and phosphatase inhibitors) for 20 min at 4°C. They were then centrifuged at 12000 rpm for 10 min at 4°C, and 2x denatured protein loading buffer was added to the supernatant. The mixture was boiled at 100°C for 10 min and cooled at room temperature. Equal amounts of protein (30–60 μg) were run on an 8–10% SDS-PAGE gel. After protein separation, they were transferred to nitrocellulose membranes. The primary antibody dilutions were: 1:1000 for both PKA and p-PKA, and 1:500 for Ki67.

### Measurement of VSMC Proliferation With Cell Counting Kit-8 (CCK8)

Cell proliferation was measured according to the reference (Liu et al., 2014). By using a CCK-8 kit, A7r5 cells were first seeded in 96-well plates at 2 × 10^3^ cells / well, and divided into seven groups: (1) blank control (cell-free medium); (2) control (cell-containing medium); (3) treated with 50 ng/ml PDGF-BBC for 24 h; (4) pretreated with 200 μM SO_2_ donor for 30 min, then 50 ng/ml PDGF-BB for 24 h; (5) treated with 200 μM SO_2_ donor for 30 min; (6) treated with 50 ng/ml PDGF-BB and 30 μM DIDS for 24 h; and (7) pretreated with 200 μM SO_2_ donor for 30 min, then 50 ng/ml PDGF-BB and 30 μM DIDS for 24 h. Ten μl of CCK8 reagent was added into a 100 μl- medium, and incubated at 37°C for 4 h. The absorbance was measured at 450 nm using a microplate reader. The mean absorbance value of the blank control group was subtracted from each group as the corrected absorbance of each group.

### Measurement of AE2 Sulphenylation in VSMCs

Sulphenic acid modification of AE2 was measured as described previously (Chen et al., 2017). The cells were divided into three groups: untreated, treated with 200 μM SO_2_ donor for 10 min, or simultaneously with SO_2_ donor and 0.4 mM DTT for 10 min. Cells were pretreated with 50 ng/ml PDGF-BB for 1 h to induce cell proliferation. DAz-2 is a specific sulphenic acid probe used to label proteins modified by sulphenic acid. The cells were lysed with non-denaturing lysis buffer containing 5 mM DAz-2 and centrifuged at 16000 *g* for 4 min at 4°C. The supernatant was divided into two parts. 10 μl was used as total protein, which was added to denatured protein loading buffer and boiled for 10 min at 100°C. The remaining supernatant was incubated at 37°C for 2 h with gentle shaking to extract the sulphenic acid modified protein. The mixture was then incubated with 250 μM p-biotin for 2 h at 37°C with gentle shaking to label the protein with biotin. Biotinylated proteins were precipitated with UltraLinkTM Immobilized NeutrAvidinTM (Thermo Fisher Scientific, Waltham, MA, United States) and incubated for 4 h on a shaker at 4°C. The beads were washed three times with PBS, and non-denatured protein loading buffer was added and boiled at 100°C for 10 min. Total protein and sulphenic acid modified protein were subjected to WB analysis. The primary antibody for AE2 was diluted 1:500. The secondary antibody was diluted 1:2000.

### Imaging the Intracellular Calcium in VSMCs

The intracellular calcium in VSMCs was imaged with fluorescent calcium probe, Fluo 4-AM. A7r5 cells were divided into four groups: PDGF-BB, PDGF-BB + SO_2_, PDGF-BB + Bay K8644, and PDGF-BB + Bay K8644 + SO_2_. Cells were incubated with 50 ng/ml PDGF-BB for 1 h, 1 μM Bay K8644, an L-type calcium channel agonist, for 30 min, and 200 μM SO_2_ for 10 min. After the treatment, the cells were washed with Krebs’ buffer for twice and incubated with 5 μM Fluo 4-AM in the dark for 30 min at 37°C. The unincorporated dye was removed by washing the cells twice. Loaded cells were maintained at room temperature for another 30 min to allow Fluo 4-AM to de-esterify. Fluorescence image was obtained using a laser scanning confocal microscope (Olympus), at appropriate wavelength settings (excitation at 488 nm and emission at 520 nm).

### Statistical Analysis

Data were processed using SPSS 17.0 software (SPSS Inc, Chicago, IL, United States). All data were expressed as mean ± standard error. To examine the effect of SO_2_ on AE, a *t*-test was performed to compare the difference between control and DIDS. To examine the effect of different concentrations of SO_2_ on AE, PKA phosphorylation and A7r5 cell proliferation, comparisons of three or more groups were analyzed by ANOVA, and the Bonferroni test or the Dunnett T3 test were used to compare the difference between two groups. *p* < 0.05 was considered statistically significant.

## Results

### SO_2_ Reduced pH_i_ in VSMCs

**FIGURE 1:**
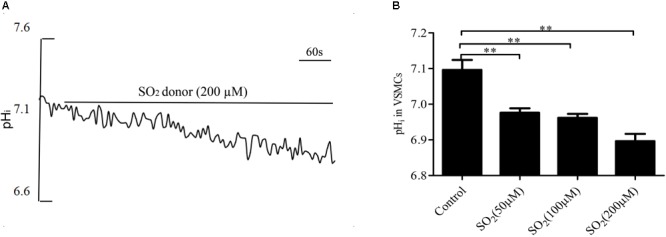
SO_2_ decreased the pH_i_ in VSMCs. **(A)** Representative tracing showing 200 μM SO_2_ donor. **(B)** Effect of SO_2_ donor on pH_i_ (*n* = 16–33). ^∗∗^*p* < 0.01 compared with pH_i_ before SO_2_ donor application.

By real-time monitoring of the pH_i_ with the fluorescent probe, we found that the SO_2_ donor at the concentrations of 50, 100, and 200 μM decreased the pH_i_ in VSMCs by 0.120 ± 0.012, 0.134 ± 0.011, and 0.200 ± 0.020, respectively (all *p* < 0.01, Figure 1A,B).

### SO_2_ Activated AE to Reduce pH_i_ in VSMCs

**FIGURE 2:**
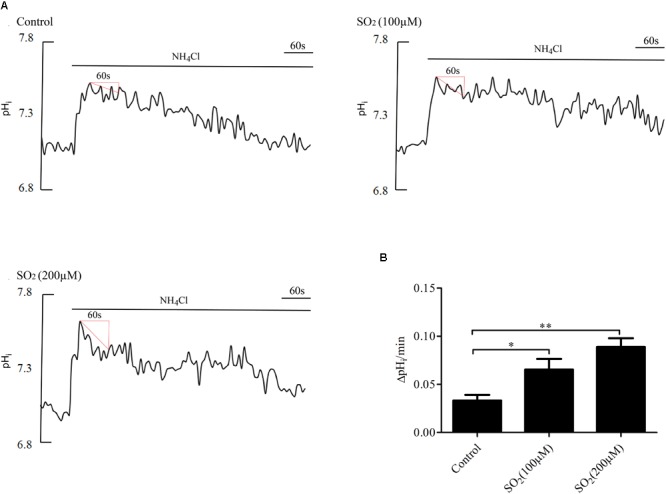
Effect of SO_2_ on AE activity. **(A)** Representative tracings of the rapid alkalinization of 20 mM NH_4_Cl and recovery from higher pH_i_ at different concentrations. SO_2_ was given 10 min before measurement. **(B)** Results were shown in mean ± SEM (*n* = 25–39). ^∗^*p* < 0.05, ^∗∗^*p* < 0.01 compared with control.

Anion exchanger is the main acid loader of VSMCs, which pumps out one HCO_3_^-^ in exchange for one Cl^-^ into the cells, maintaining intracellular Cl^-^ concentration and lowering the pH_i_. To further validate the effect of SO_2_ on the AE, we tested the activity of the AE in VSMCs by using the widely accepted NH_4_Cl perfusion method. As shown in Figure 2A,B, the SO_2_ donor at varying concentrations of 100 and 200 μM activated the AE in VSMCs (*p* < 0.05 and *p* < 0.01, respectively).

**FIGURE 3:**
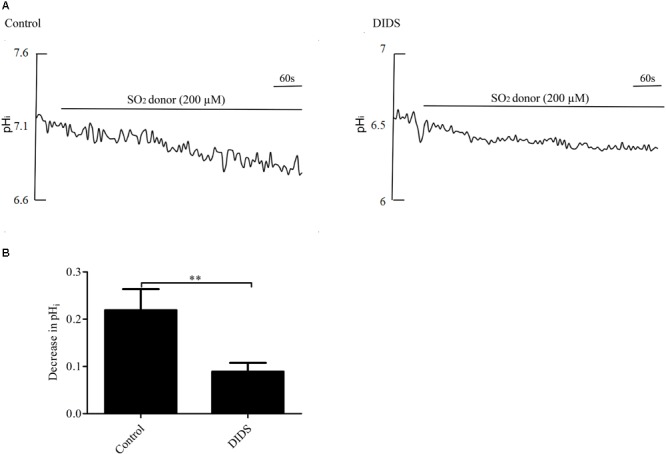
Effect of SO_2_ on pH_i_ in the presence or absence of DIDS in VSMCs. **(A)** Representative tracings of 200 μM SO_2_ donor alone (*n* = 19) and in the presence of 30 μM DIDS (*n* = 19). **(B)** Results were shown in mean ± SEM. ^∗∗^*p* < 0.01 compared with control.

Pretreatment with 30 μM DIDS, a Cl^-^/HCO_3_^-^ exchanger inhibitor, for 20 min significantly attenuated the pH_i_ reduction caused by SO_2_ (*p* < 0.01, Figure 3A,B), suggesting that SO_2_ donor reduced pH_i_ by activating the AE in VSMCs.

### SO_2_ Activated AE by Sulphenylating AE2 in VSMCs

**FIGURE 4:**
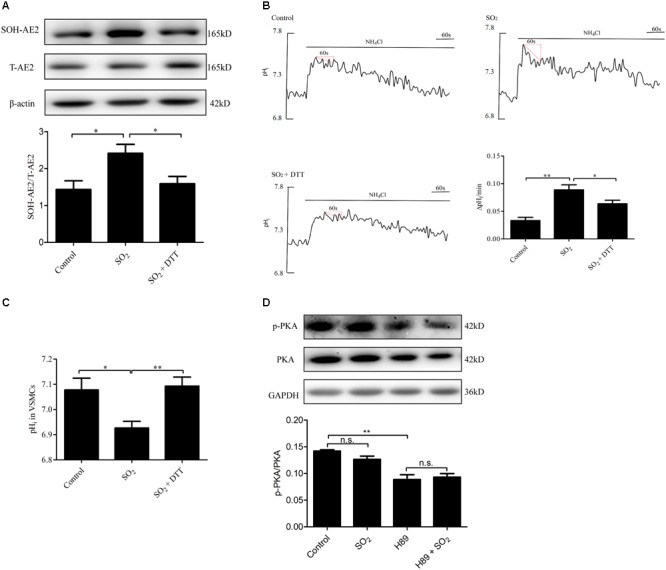
Mechanisms by which SO_2_ donor activated AE. **(A)** Sulphenylation of AE2 by 200 μM SO_2_, and 0.4 mM DTT reversed the process. SO_2_ and DTT were given 10 min before collecting cells (*n* = 6). **(B)** Representative tracings of AE activity in the absence or presence of 200 μM SO_2_ donor and 0.4 mM DTT. Results were shown as mean ± SEM (*n* = 25–39). **(C)** The change of pHi in VSMCs in each group. **(D)** Effect of SO_2_ donor on PKA phosphorylation. Cells were pretreated with H89 30 min, followed by SO_2_ donor in 10 min (*n* = 6). ^∗^*p* < 0.05, ^∗∗^*p* < 0.01, n.s.*p* > 0.05.

To investigate the mechanism by which SO_2_ activates AE activity in VSMCs, we tested whether SO_2_ oxidizes Cys of AE2 by AE2 sulphenylation. As shown in Figure 4A,B, SO_2_ promoted AE2 sulphenylation and AE activation (*p* < 0.05, *p* < 0.01, respectively), and reduced pH_i_ (*p* < 0.05). Addition of 0.4 mM DTT, a thiol reductant, reversed SO_2_-induced AE2 sulphenylation and activation of the AE (both *p* < 0.05), restoring SO_2_-induced pH_i_ reduction in VSMCs as well (*p* < 0.01, Figure 4C). This suggests that the thiol group is a likely target of SO_2_ for activation of the AE in VSMCs.

We also aimed to understand if SO_2_ affects the pH_i_ by activation of PKA and further activation of the exchanger in VSMCs. As shown in Figure 4D, SO_2_ (200 μM) did not stimulate PKA within 10 min in VSMCs (*p* > 0.05). The results suggested that the PKA pathway did not mediate pH_i_ reduction by SO_2_ in an acute phase.

### SO_2_ Inhibited VSMC Proliferation Depending on AE Activation

**FIGURE 5:**
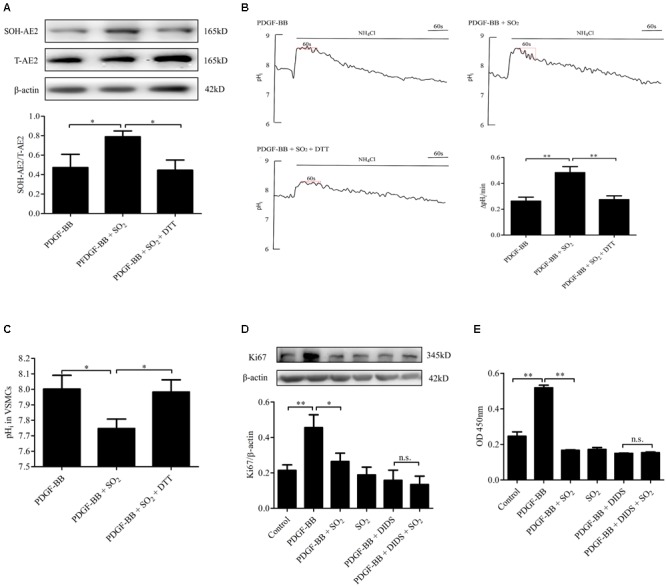
Activation of AE was responsible for SO_2_ to inhibit VSMC proliferation. **(A)** Sulphenylation of AE2 by SO_2_ in the presence of PDGF-BB. Cells were pretreated with 50 ng/ml PDGF-BB 1 h, followed by 200 μM SO_2_ and 0.4 mM DTT 10 min (*n* = 6). **(B)** Representative tracings of AE activity in the absence or presence of 200 μM SO_2_ donor and 0.4 mM DTT when cells were pretreated with 50 ng/ml PDGF-BB 1 h (*n* = 31–39). **(C)** pH_i_ change in each group. **(D,E)** WB and CCK8 were used to measure the proliferation of VSMCs. 50 ng/ml PDGF-BB, 200 μM SO_2_ and 30 μM DIDS were administered for 24 h (*n* = 6). ^∗^*p* < 0.05, ^∗∗^*p* < 0.01, n.s.*p* > 0.05.

To further elucidate if SO_2_ inhibit VSMC proliferation depending on AE activation, we constructed a PDGF-BB-induced VSMC proliferation cell model. In VSMCs pretreated with PDGF-BB and treated with SO_2_, levels of AE2 sulphenylation and AE activity were significantly increased (*p* < 0.05, *p* < 0.01, respectively, Figure 5A,B) and the pH_i_ was significantly reduced (*p* < 0.05, Figure 5C). VSMC proliferation evaluated by Ki67 protein expression and CCK8 activity was significantly inhibited by SO_2_ (*p* < 0.05, *p* < 0.01, respectively, Figure 5D,E), as compared with those in VSMCs pretreated with PDGF-BB only. However, SO_2_ failed to inhibit PDGF-BB-induced VSMCs proliferation once AE was inhibited by DIDS (both *p* > 0.05, Figure 5D,E).

### SO_2_ Reduced the Intracellular Calcium by Inhibiting L-Type Calcium Channel in VSMCs

**FIGURE 6:**
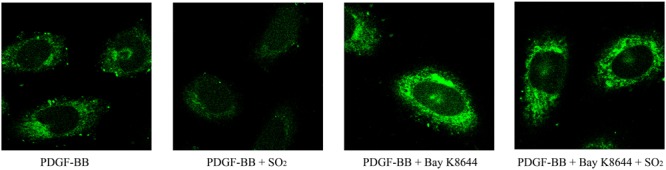
SO_2_ decreased the intracellular calcium level by inhibiting L-type calcium channel in VSMCs. Cells were incubated with 50 ng/ml PDGF-BB for 1 h, 1 μM Bay K8644 for 30 min and 200 μM SO_2_ for 10 min. Fluorescence intensity represents calcium levels.

To investigate the effects of SO_2_ on intracellular calcium in VSMCs, we used Fluo 4-AM to image the intracellular calcium in VSMCs. The results showed that SO_2_ reduced PDGF-stimulated intracellular calcium in VSMCs. While, Bay K8644, an L-type calcium channel agonist, blocked SO_2_-reduced intracellular calcium content in PDGF-stimulated VSMCs (Figure 6). The results indicated that SO_2_ might decrease the calcium level by inhibiting L-type calcium channel in VSMCs.

## Discussion

As well known, pH_i_ is precisely controlled and needs to be maintained within physiological range. The imbalance of pH_i_ is an important pathological basis for the abnormal cell metabolism and life activities. Therefore, pH_i_ is a vital target for clinical treatment of diseases. In the present study, we firstly reported that SO_2_ donor decreased the pH_i_ of VSMCs by enhancing AE2 sulphenylation to activate the AE, which might partially mediate the inhibitory effect of SO_2_ on VSMC proliferation.

At first, by monitoring pH_i_ in real time, we found that the treatment of 50 ∼ 200 μM SO_2_ donor for 9 min decreased pH_i_ in VSMCs by 1.7–2.8%, respectively. The mechanisms by which SO_2_ reduced pH_i_ in VSMCs, however, have yet been unclear. AE is the main acid loader in VSMCs and plays an important role in the regulation of pH_i_ (Vigne et al., 1988). Previous studies reported that pH_i_ was increased significantly when NH_4_Cl loaded cells, and consequently AE was activated to extrude HCO_3_^-^, which eventually restored the pH_i_ to normal levels (Xu and Spitzer, 1994; Lee et al., 2007). Therefore, we directly detected the activity of AE using NH_4_Cl stimulation as previously reported (Simchowitz and roos, 1985). The data showed that SO_2_ donor markedly steepened the recovery slope of pH_i_ trace following the pH_i_ peak due to NH_4_^+^-induced alkalinization, suggesting that SO_2_ activated AE in VSMCs as we expected. Subsequently, the fact that an inhibitor of Cl^-^/HCO_3_^-^ exchanger, DIDS blunted the SO_2_-induced decrease in pH_i_ in VSMCs supported the speculation that the activation of AE was involved in the effect of SO_2_ on the pH_i_ in VSMCs.

However, up to now, the mechanisms by which SO_2_ activated the AE in VSMCs remain unclear. Cysteine thiol group (-SH) can be oxidized to sulphenic acids (-SOH) and the process is termed as protein sulphenylation, which is an important mechanism for regulating protein function. Hourihan et al. (2016) found that sulphenylation of inositol-requiring enzyme 1 could inhibit endoplasmic reticulum stress and activate antioxidant response. The sulphenylation of NF-κB p65 by SO_2_ resulted in an inactivation of NF-κB pathway (Chen et al., 2017). While Cys residues mutation within transmembrane domain of AE2 could affect the activity of AE2 (Reimold et al., 2013). Interestingly, our data showed that SO_2_ sulphenylated AE2 in VSMCs in association with the enhancement of the activity of AE. While, a thiol reductant DTT blocked the effect of SO_2_ on sulphenylation and the activity of AE2. Moreover, PKA pathway was also reported to participate the activation of AE (Puceat, 1999) and SO_2_ treatment for 30 min activated cAMP/PKA pathway (Liu et al., 2014). But, in the present study, SO_2_ incubation for 9 min could not enhance the phosphorylation of PKA, which excluded the possibility that SO_2_ indirectly promoted the activation of AE via PKA pathway. Those above results suggested that SO_2_ might directly activate the AE by inducing the sulphenylation of AE2 at the posttranslational level.

To investigate the significance of SO_2_-induced reduction of pH_i_ in VSMCs in its inhibitory effect on VSMC proliferation, DIDS was used to block the SO_2_-induced reduction of pH_i_ in VSMCs, and then Ki67 expression and CCK8 activity were analyzed as the markers of VSMC proliferation. We found that SO_2_ inhibited PDGF-BB-induced VSMC proliferation, while DIDS abolished the inhibitory effect of SO_2_ on PDGF-BB-induced VSMC proliferation, which suggested that SO_2_ inhibited VSMC proliferation at least partly through decreasing pH_i_ in VSMCs.

However, we also found an interesting detached phenomenon that SO_2_ donor alone did not inhibit the proliferation of VSMC but inhibited PDGF-BB-induced VSMC proliferation. In fact, under physiological condition, VSMC-derived endogenous SO_2_ was sufficient enough to inhibit cell proliferation. Therefore, the supplement of additional exogenous SO_2_ donor to the VSMC on the basis of sufficient endogenous SO_2_ level would not further exert the anti-proliferative effect (Liu et al., 2014). However, under certain pathophysiological conditions, when VSMCs were insulted by the exogenous injury stimuli, endogenous SO_2_ production was decreased and the anti-proliferative effect was weakened, resulting to the excessive cell proliferation. In such a case, the supplement of SO_2_ donor, on the basis of the deficient endogenous SO_2_ level, would exert a markedly anti-proliferative effect on the proliferating VSMC (Sun et al., 2010; Liu et al., 2014; Wu et al., 2016; Yu et al., 2016). In brief, this discrepancy effect of SO_2_ donor on the VSMC proliferation on the different conditions might provide a novel idea for the treatment of vascular remodeling in vascular-injury diseases.

In addition to pH_i_, Ca^2+^ mobilization is another important stimulus for cell migration and proliferation (Yamamura, 2014; Luo et al., 2018). Endogenous SO_2_ and its derivates could inhibit L-type calcium channel, which might help explain the mechanism of vasorelaxant function (Du et al., 2008). Therefore, we observed the effect of SO_2_ on the L-type calcium channel. The data showed that 200 μM SO_2_ could inhibit the PDGF-BB-stimulated increase in the cytosolic Ca^2+^ concentration in VSMCs. However, pretreatment of Bay K8644, a specific activator of L-type calcium channel, could block the inhibitory effect of SO_2_ on the cytosolic Ca^2+^ concentration, suggesting that L-type calcium channel inhibition occurs under experimental conditions in the presence of SO_2_ donor.

However, our study still has some limitation. For example, we studied the effect of SO_2_ on pH_i_ only in cultured VSMCs, but whether the phenomenon occurs in the complex vascular wall is still unknown. As we all know, vascular wall is composed of complex multicellular tissue. It needs to respond to various stimuli such as mechanical stress and neurological and humoral factors in a coordinate manner. The intercellular communication among the constituent cells of vessel wall plays an important role in the regulation of vascular structure and activity and is indispensable for the synchronous response by the wall of vessels. The gap junction was found to exert the abovementioned function of intercellular communication (Haefliger et al., 2004; Sorensen and Holstein-Rathlou, 2012; Yang et al., 2017). The gap junction is composed of a kind of transmembrane proteins termed connexin and form a direct conduit for the exchanges of intercellular signals such as ions and bioactive metabolites, which allows vessel cells to sense the functional and metabolic state of neighbor cells and rapidly modulate the activity by themselves, and therefore synchronously respond to the stimuli (Haefliger et al., 2004; Sorensen and Holstein-Rathlou, 2012; Yang et al., 2017). In addition to the gap junction, gasotransmitters also participate the intercellular communication. Since 1980s, endothelial nitric oxide as the first gasotransmitter was found to regulate the VSMC relaxation via a paracrine pathway, which is partly due to its unique properties including small molecular weight, rapid transmembrane diffusion and extensive action (Wang, 2014; Huang et al., 2016; Kimura, 2016; Nagpure and Bian, 2016). In the previous study, pulmonary artery smooth muscle cell-derived SO_2_ was found to inhibit the collagen accumulation in the pulmonary artery fibroblasts (Yu et al., 2016). Therefore, we speculated that SO_2_ might act as an intercellular signal molecule to transduce the massagers among the constitute cells in the vessels, which participated in the synchronicity of vascular function. However, more experiments are needed to extend the effect of SO_2_ on single channel activity to complex vascular wall.

## Conclusion

We discovered the effect of SO_2_ on pH_i_ of VSMCs and clarified the mechanism by which SO_2_ decreased pH_i_ of VSMCs. Most importantly, we demonstrated that SO_2_-induced decrease in pH_i_ of VSMCs might participate the inhibitory effect of SO_2_ donor on the VSMC proliferation stimulated by mitogen such as PDGF-BB. We expect that those interesting results maybe provide a new idea for the potential clinical prevention and treatment of vascular remodeling in vascular injury diseases such as hypertension.

## Data Availability

All datasets generated for this study are included in the manuscript and/or the Supplementary Files.

## Author Contributions

YW, JD, and HJ designed the study. YW performed the experiments, analyzed the data, and wrote the manuscript. HJ, JD, and SC revised the manuscript. CT and YH provided useful suggestion and comments to the design of the research. XW, XT, and LZ were involved in the experiments. All the authors read the manuscript and approved the final version.

## Conflict of Interest Statement

The authors declare that the research was conducted in the absence of any commercial or financial relationships that could be construed as a potential conflict of interest.

## Supplementary Material

FIGURE S1 The fluorescence intensity-pH_i_ standard curve obtained with nigericin.
