# Supplementary figures and images for: Sulfur Dioxide Activates Cl-/HCO3- Exchanger via Sulphenylating AE2 to Reduce Intracellular pH in Vascular Smooth Muscle Cells
Source: Front Pharmacol. 2019 Mar 27;10:313. doi: 10.3389/fphar.2019.00313 (PMC6446831; doi:10.3389/fphar.2019.00313)

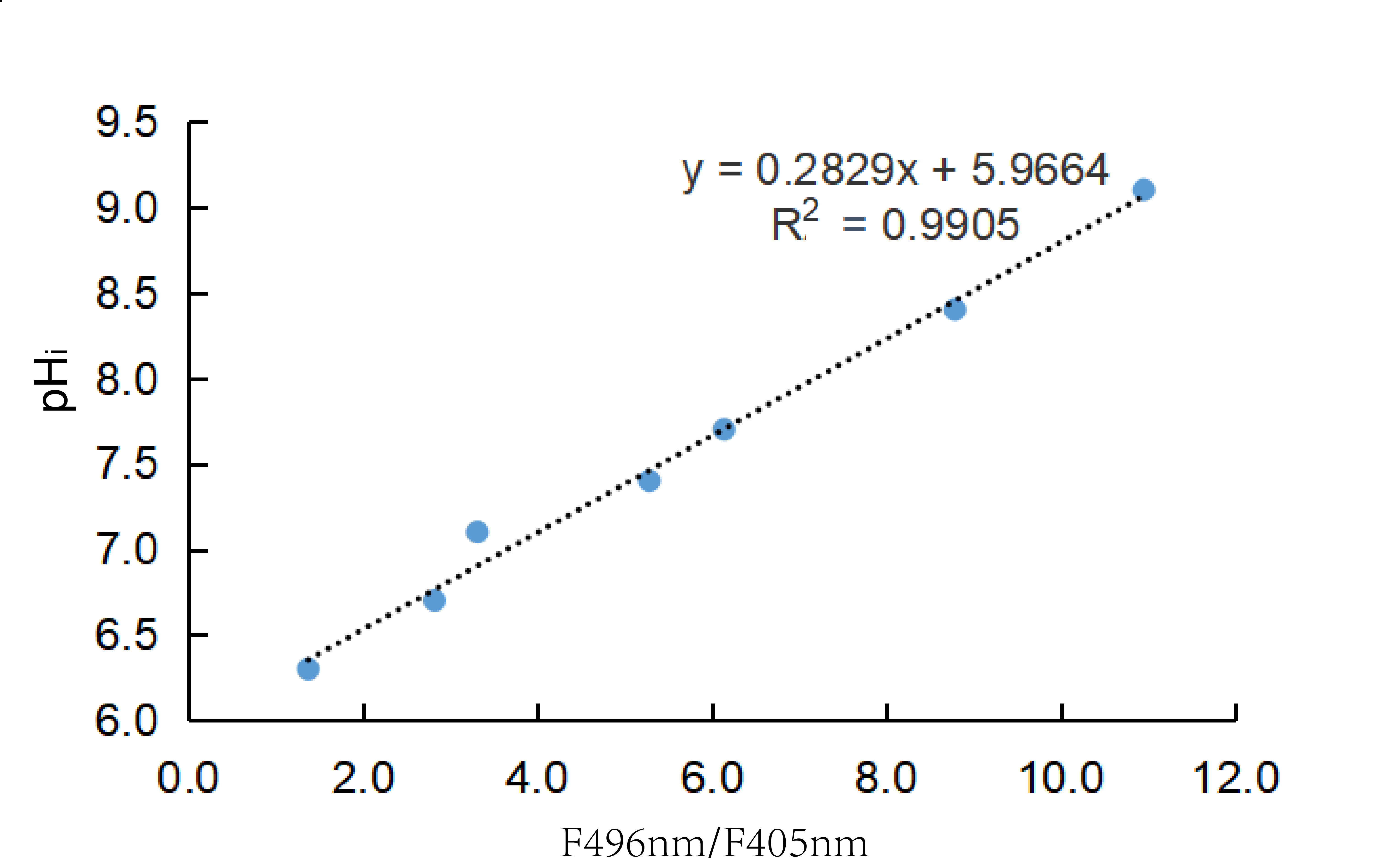

Supplement: FIGURE S1 — The fluorescence intensity-pHi standard curve obtained with nigericin. [file Image_1.JPEG]
